# Numerical Approach to Simulate the Mechanical Behavior of Biodegradable Polymers during Erosion

**DOI:** 10.3390/polym15091979

**Published:** 2023-04-22

**Authors:** André F. C. Vieira, Enio H. P. Da Silva, Marcelo L. Ribeiro

**Affiliations:** 1 Center for Mechanical and Aerospace Science and Technologies (C-MAST-UBI), Universidade da Beira Interior, R. Marquês D’Ávila e Bolama, 6201-001 Covilhã, Portugal; 2 Aeronautical Engineering Department, São Carlos School of Engineering, University of São Paulo, São Carlos 13563-120, SP, Brazil

**Keywords:** biodegradable polymers, hydrolytic damage, polymer erosion, finite element model

## Abstract

Biodegradable polymers find applications in many market segments. The ability to meet mechanical requirements within a certain time range, after which it degrades and is naturally absorbed, can be used to produce short-term use products that can be easily disposable with less environmental impact. In the segment of medical devices used in regenerative medicine, these materials are used to produce temporary implants that are naturally assimilated by the human body, avoiding a removal surgery. However, the design of these temporary devices still presents great challenges, namely in the verification of the main requirement: the lifetime of the device, associated with the progressive loss of mechanical properties, until its complete erosion and assimilation. Thus, in this study, a numerical approach is proposed to simulate the polymeric device’s mechanical behavior during its hydrolytic degradation by combining the hydrolysis kinetics, that depends on mechanical factors and promotes a decrease of molecular weight and consequent decrease of mechanical performance, and erosion, when molecular weight reaches a threshold value and the polymer becomes soluble and diffuses outward, resulting in mass loss and decreasing cross-sectional area, which also contributes to the mechanical performance reduction of the device. A phenomenological approach, using the combination of continuum-based hydrolytic damage for the evolution of mechanical properties that depends on the stress field and further removal of the degraded element (to simulate mass loss) was used. Both elastoplastic and hyperelastic constitutive models were applied on this study, where the material model parameters locally depend on the molecular weight.

## 1. Introduction

Biodegradable polymers find applications in many market segments. Among its main advantages is the ability to meet mechanical requirements within a certain time range, after which it degrades and is naturally absorbed. Short-term use products can be easily disposed of, saving significantly on waste removal. Typical examples of applications of these materials are fishing nets or agricultural devices used during one season. Products made from biodegradable plastics have an environmental advantage in various short-term applications, which can be clearly demonstrated through a life cycle assessment. On the one hand, they are obtained from renewable resources, unlike conventional thermoplastics obtained from petroleum. The monomers are obtained, after processing, from agricultural products (such as sugar, potatoes, soybeans, corn, etc.) or from genetically modified bacteria cultivated in optimized environments. Thus, its degradation in a composting environment or incineration results in CO${}_{2}$ emissions that were naturally accumulated by photosynthesis and that would occur anyway with the degradation of the source plants.

In the segment of medical devices used in regenerative medicine, these materials are, in many cases, biocompatible. Hence, they can be used in combination with cells to produce temporary implants that replace the mechanical functions of the extracellular matrix while the biological tissue gradually regenerates and replaces the implant that degrades and is naturally assimilated by the human body. This way, implant removal surgeries are avoided. These types of solutions can in many situations replace transplants, autografts, or xenografts.

However, the design of these temporary devices still presents great challenges, namely in the verification of the main requirement: the lifetime of the device, associated with the progressive loss of mechanical properties, until its complete erosion and assimilation. The performance of a biodegradable polymeric part and its compliance with functional requirements can be assessed experimentally by trial-and-error experiments in the same environmental conditions as service situations. However, these experiments, usually applied in the design phase to find the correct dimensions of the devices that enable their compliance, take a lot of time and resources. Hence, a digital twin can be applied to iteratively compute the device dimensioning. Thus, in this study, a numerical approach is proposed to simulate the polymeric device’s mechanical behavior during its hydrolytic degradation by combining the hydrolysis kinetics, which depends on mechanical factors and promotes a decrease in molecular weight and consequent decrease in mechanical performance, and erosion, when molecular weight reaches a threshold value and the polymer becomes soluble and diffuses outward, resulting in mass loss and decreasing cross-sectional area, which also contributes to the mechanical performance reduction of the device.

### 1.1. Kinetic Models for Erosion and Degradation

In applications under aqueous environments, such as implants or devices submerged in water, an important distinction must be made between erosion and degradation. Erosion is related to mass loss; hence, it is measured by comparing the dry weight of specimens before and after a certain incubation time. On the other hand, degradation is related to a decrease in molecular weight due to hydrolytic reactions that occur in the presence of water or enzymes. Hence, it can be estimated by measuring the molecular weight of specimens before and after a certain incubation time using size exclusion chromatography (SEC) or gel permeation chromatography (GPC). Furthermore, molecular weight is directly related to polymer strength, and therefore hydrolysis promotes a decrease of mechanical performance (strength and toughness reduction), which can be estimated by mechanical testing. The erosion process can be described by phenomenological diffusion-reaction mechanisms. Initially, in contact with the degradation environment, while water diffuses more rapidly into the material, enzymes, since they are large molecules, are unable to do it, and so they degrade at the surface. Their presence triggers the hydrolytic reaction, leading to the scission of macromolecules and the creation of smaller fragments called oligomers. Furthermore, when the polymeric chain fragments are small enough to be soluble on the aqueous media, oligomeric products can diffuse outwards to be then bio-assimilated by the host environment. For example, PLA becomes soluble in water at a molecular weight, Mn, below ≈20.000 (g/mol) [1]. This way, the material loses mass. The complete erosion of the polymer is known to take substantially longer than the complete loss of strength.

Several factors affect the local degradation rate. Hence, hydrolysis rates may evolve heterogeneously along the volume due to heterogenous scalar fields, such as temperature, concentration of water/enzyme or pH of the degradation media, or tensor fields, such as stress or strain. Several works reported that higher temperature increases the hydrolytic degradation rate [2,3,4]. Other works have reported the influence of mechanical stress (or strain) on the hydrolytic degradation rate [5,6,7,8,9,10]. Analogously to the temperature, stress (or strain) applied during polymer degradation will increase the probability of bond scission and therefore the hydrolysis rate. Some other works also report that the hydrolytic degradation rate is influenced by the presence of certain enzymes [11,12] or by the pH of the aqueous medium [13,14,15].

Another important consideration is that the formation of oligomers decreases the local pH. Hence, this leads to a local increase in the hydrolysis rate due to oligomer retention before they can diffuse outward and the formation of hollows inside. This phenomenon is called autocatalysis. It was observed, both in vitro [16] and in vivo [17], that hydrolytic degradation evolves heterogeneously along the volume in the case of large specimens made of aliphatic polyesters derived from lactic and glycolic acids (PLA/GA polymers). A faster degradation in the core was found compared to that at the surface [16,18,19,20]. In the long term, thick specimens eroded faster than the thinner ones made of the same polymer under the same environmental conditions. The crystalline degree is another crucial factor since diffusion is more difficult in these regions.

The penetrating water rapidly creates a gradient of water concentrations from the surface to the center. However, this gradient vanishes in a couple of hours or days when the specimen saturates. Depending on the polymer hydrophobicity, the diffusion rate can be higher or lower than the hydrolysis rate. If diffusion is very slow in comparison to the hydrolysis reaction, or if the specimen is too thick, water onset at the core of the specimen is delayed and hydrolysis occurs mainly at the surface. For example, enzymatic degradation also fits this type of surface degradation since enzymes are large molecules, which make it difficult to diffuse them towards the specimen’s core. On the other extreme, we have bulk degradation, when water diffusion is considerably faster than hydrolysis. In this case, water reaches the specimens core at nearly the same time it reaches the surface. This is the case for thin specimens or specimens with large open porous. In this case, water can be assumed to have been uniformly distributed along the volume from the first instant of incubation. If saturation is considered instantaneous (i.e., no diffusion control), hydrolysis will start homogeneously along the specimens volume. Assuming that water concentration, temperature, stress field, and degradation medium are constant during degradation along the volume of an amorphous polymer, then the hydrolytic rate will be constant and homogeneously distributed during incubation time. For all polymers, water absorption leads to further recrystallization. Furthermore, water acts as a plasticizer, softening the material and lowering the glass transition temperature. Since a highly hydrophilic polymer, such as a hydrogel, becomes more ductile when saturated, the constitutive relationship should also depend on water concentration (from dry to wet). However, in this study, we will consider the constitutive relations of wet polymers. This implies that mechanical tests used to calibrate the material parameters of constitutive models should be performed on wet specimens.

Random scission hydrolysis is the simplest model to describe the hydrolysis kinetics. The increase of carboxylic acid end groups is described by a first-order differential equation. This is a very simple approach to computing the molecular weight evolution. This simple model also disconsidered the local decrease in pH and the corresponding accelerated degradation at the core due to the presence of the newly formed carboxylic acid end groups. Nor can I describe the recrystallization effect promoted by the increased mobility of the smaller polymer chains. To consider a semicrystalline polymer, distinct degradation rates should be used for amorphous and crystalline regions.

To describe the erosion kinetics and compute mass loss, the diffusion of oligomers outwards should also be considered. More advanced models were developed over the past year to predict the evolution of molecular weight along with volume and mass loss. These models can be classified as empirical or phenomenological. In both classes, there are also deterministic and stochastic models. Autocatalytic hydrolysis, oligomer production, and their diffusion, together with erosion (bulk and surface) using the Monte Carlo modeling methods and a set of differential equations were considered in a recent erosion model proposed by Sevim and Pan [21] In another work of Zhang et al. [22], recrystallization induced by polymer chain scissions, was also considered. This complicated multi-scale model uses cellular automata and Monte Carlo methods to consider the interactions between microscopic hydrolysis, the mesoscopic formation of cavities due to autocatalysis, and the macroscopic diffusion of oligomers. These recent models allow for the heterogeneous evolution of molecular weight, crystallinity, and mass during incubation time, enabling the understanding of transitions between bulk and surface erosion. However, none of these models was able to predict the mechanical behavior evolution of the device or its compliance with functional mechanical requirements. They are used, for example, to predict the drug delivery kinetics, but are unable to predict the mechanical performance reduction. For that purpose, these models must be combined with constitutive models. On the one hand, the volume loss due to erosion determines a reduction in the mechanical performance of the device, but the reduction in strength and shear modulus of the remaining volume also contributes to this reduction in mechanical performance.

### 1.2. Constitutive Models for Polymers

Loads acting on solids lead to changes in their original shape, which causes stress fields. Constitutive models are used to describe the relation between a given stress field and the strain field for any point of the solid. Constitutive models can be classified as time-independent or time-dependent models. Time-independent models enable us to represent the relaxed configuration of a solid, considering that a load will immediately change the solid’s shape. Therefore, this class of models are used to represent quasi-static loading case scenarios. Time-dependent models consider an evolution of the shape after loading, therefore enabling the representation of transient configurations. The simple and classic linear elastic model, known as the Hooke’s model, is a time independent model commonly used to simulate the mechanical behavior of metals bellow yielding stress. In most cases, the mechanical simulation of biodegradable devices considers an elastic or elastoplastic behavior, neglecting any changes on the mechanical behavior due to molecular weight reduction or volume decrease [23]. In many applications, such as a biodegradable polymeric stent or a ligament, the biodegradable polymeric structures are submitted to cyclic loading above yielding. Hence, they are prone to accumulate plastic strain at each cycle, which may lead to progressive laxity and consequent failure of the device. In addition to the non-linear nature of polymers, their mechanical behavior is also time-dependent at room temperature, unlike metal or ceramics. The mechanical behavior of polymers depends on the strain rate, and time independent models will simulate the same mechanical behavior for different strain rates. While at low strain rates, its behavior is ductile, at high strain rates, it changes to brittle behavior [24]. When a constant load (below its strength) is applied to a generic polymer at room temperature, its shape will progressively change until it collapses when a critical strain is reached. This creep phenomenon is due to the viscous flow of polymeric chains. Grabow et al. [25] reported a significant susceptibility of polylactides to creep. Vieira et al. [25] also reported that biodegradable polymers exhibit hysteresis, i.e., a different stress-strain path between loading and unloading that corresponds to energy dissipation in the form of heat. To simulate those time-dependent phenomena, time-dependent models are required. Dissipative elements representing time inhomogeneous relations must be used in the model formulation to consider time dependency.

The mechanical behavior of polymers, under a generic loading spectrum where large deformations and dynamic loading at varying strain rates are present, combines the elastoplastic behavior, typical of metals, and the viscous behavior typical of fluids. Using the Boltzmann superposition principle, hyperelastic, plastic, and viscous models can be combined to describe this mechanical behavior. Hence, more complex viscoplastic constitutive models with several material parameters are needed. An extensive set of experimental tests, combining tests at different strain rates, creep tests, relaxation tests, load-unloading tests, among others, are needed to calibrate the material model parameters of these viscoplastic models. Examples of these viscoelastic and viscoplastic constitutive models can be found in literature [24,26,27,28,29]. Despite this, those models are used to simulate the behaviour of non-degradable polymers and don’t consider the time evolution of the mechanical behavior while the molecular weight is reducing. This can be accomplished when the material model parameter evolution during incubation is related to the hydrolysis kinetics and the molecular weight evolution.

### 1.3. Approaches to Simulate the Mechanical Behaviour during Hydrolytic Degradation

Only a few scientific contributions about the modeling of the mechanical behavior of biodegradable polymers during the hydrolytic degradation process can be found in the literature [5,30,31,32,33,34,35,36,37,38,39]. These can be divided into two types of approaches. In the first one, the constitutive relations are maintained, but the material model parameters of constitutive models are function of a scalar field, which represents the local chemical damage due to hydrolysis and the corresponding molecular weight reduction. Among this kind of approach, hyperelastic models [36,37], quasi-linear viscoelastic models [34], and viscoplastic models [33,38] can be found in the literature. In some of these models, the local degradation rate depends on local variables such as water concentration and stress state. These local variables are heterogeneous first- or second-order tensor fields. The development of numerical models based on the finite element method can then allow the simulation of the structural mechanical behavior of a polymeric device with generic geometry and boundary conditions at a given incubation time. However, on this kind of approach, the volume remains unchanged, i.e., the performance reduction is only due to a molecular weight decrease. On the second type of approaches [31,32,39] the linear elastic model is used, and the evolution of Young´s modulus is modeled considering chain scissions and the consequent formation of cavities and recrystallization in a Representative Volume Element (RVE) at a microscale (atomic level). These approaches can represent a significant lag between the reduction of Young´s modulus after the reduction of the molecular weight generally observed in most polymers, and the increase of Young´s modulus in the initial phase of degradation due to an increasing crystallinity. One important limitation found in these approaches [31,32,39] is that degradation kinetics do not depend on the stress field. In a recent approach presented by Guo et al. [5], elements of the finite element model become inactive when a local variable, related to mass loss, reaches a limit value. Therefore, this method is not coupled to degradation kinetics but to erosion kinetics, and material model parameters are constant during incubation time. In this approach, the mechanical performance reduction is due to a material volume decrease. However, in this work [5], the rate of mass loss is stress dependent. This approach [5] is more realistic for modeling surface erosion in polymers.

The objective of this paper is to use a simple kinetic model to predict the local molecular weight evolution and combine it with the material model parameters of elastoplastic and hyperelastic constitutive models. This will result in the decrease of shear module related to the molecular weight, in the case of an hyperelastic model or the isotropic hardening in the case of an elastoplastic model. Furthermore, when the local molecular weight reaches a threshold value, the corresponding element is erased. This will be related to the mass loss of the polymer and enable the simulation of erosion. As a result of this, the decrease in the cross-section leads to a different structural response since a smaller volume will sustain the same loads. The numerical model also accounts for the polydispersity index (PI) related to the molecular weight distribution. Furthermore, after materials and methods, where the hydrolytic damage and the erosion processes are better discussed, the implementation of the finite element model will be described in more detail. In the last part, the simulation results are presented, and the results are finally discussed.

## 2. Materials and Methods

The modeling approaches to predict degradation of polymers could be classified as either physical or phenomenological approaches. The physically-based degradation models use theoretical frameworks that capture the chemical processes taking place on polymers, and additionally, these approaches are computationally prohibitive. On the other hand, phenomenological approaches by Chen et al. [40] use combinations of continuum-based damage mechanics and/or removal of the degraded element to simulate mass loss. The last approach is applied in this work to simulate the polymer hydrolytic damage and erosion. In this work, we combine a simple phenomenological kinetic model with simple time-independent models. This simplicity introduces some limitations discussed above, but the main idea is to exemplify a possible combination between kinetic models for degradation and constitutive models that describe the mechanical behavior. More complex kinetic models or constitutive models can be used with a similar approach.

### 2.1. Kinetic Models for Erosion and Degradation

As discussed above, water absorption occurs immediately after a biodegradable polymeric structure is immersed in an aqueous medium. Diffusion of water depends on factors such as the hydrophilicity of the polymer, crystallinity, temperature, flow, and pH of the aqueous medium. Furthermore, the local presence of water triggers the hydrolysis reaction, leading to the scission of molecules. On the other hand, as will be discussed, hydrolytic rate is directly proportional to the water concentration. Fick’s Law, given by Equation (1), is commonly used to describe the evolution of water concentration *w* during incubation time *t* in a finite element volume at a distance *x* from the surface:

(1) $$\frac{dw}{dt}=D\frac{\partial^{2}w}{\partial x^{2}}$$

where *D* is the diffusion coefficient in the case of an isotropic polymer, considering that diffusion evolves equally in all directions. By measuring the evolution of weight during the incubation time, in specimens having two different thickness, the diffusion coefficient *D* can be determined via inverse calibration. In this work, we will consider a constant diffusion coefficient for a given polymer, considering a constant and homogenous crystalline degree, temperature, and pH.

As referred to above, the simplest way to describe the hydrolysis reaction is the first-order kinematic differential equation, according to the Michaelis-Menten scheme [41], where polymer scissions occur randomly at any hydrogen bound along the polymer chain. According to Laycock et al. [42], random scission is predominant in the early stages of hydrolysis, when a rapid loss of molecular weight and strength occurs. For example, in aliphatic polyesters (such as polylactides, polyglicolic acid, polycaprolactones, etc.), each polymeric chain contains an immense sequence of ester groups, and has a carboxylic radical on one end and an alcohol group on the other end. Furthermore, the hydronium ion will react with a random ester group, splitting the macromolecule into two smaller molecules. Hence, while the polymer chains are being divided, the molecular weight decreases, and the number of end groups increases inversely. This simple model describes the evolution of carboxylic end groups during degradation time (Equation (2)):

(2) $$\frac{dc}{dt}=kewc=uc$$

where *c* is the concentration of the carboxyl end groups, *t* is the degradation time, *k* is the rate constant (it is a function of the activation energy of the material, the universal constant of gases, and temperature), *e* is the concentration of ester groups, and *w* is the concentration of water in the polymer. The product *u* represents the hydrolysis reaction rate. Since the average molecular weight is the inverse of the concentration of carboxylic end groups, after integration, Equation (2) becomes:

(3) $$M_{nt}=M_{n0}e^{-u}y$$

where $M_{nt}$ is the average molecular weight at a given degradation time *t* and $M_{n0}$ is the initial average molecular weight of the polymer before degradation. As mentioned above, the logarithmic decrease in molecular weight during hydrolytic degradation results in a decrease in mechanical performance due to the hydrolytic chain scission of polymeric macromolecules. Vieira et al. [37] reported that the polymer strength follows the same logarithmic trend as the molecular weight (Equation (4)):

(4) $$S_{t}=S_{0}e^{-ut}=S_{0}e^{kwet}$$

where $S_{t}$ is the strength of the polymer at degradation time *t* and $S_{0}$ is the initial strength. In a semi-logarithmic representation of normalized strength or number-average molecular weight versus degradation time, the slope of the linear fit represents the degradation rate *u* according to Equations (3) and (4). This Equation (4) can further be used as a failure criteria. Hydrolytic damage was defined in a previous work of Vieira et al. [37] as the ratio between the initial molecular weight and the molecular weight at a given degradation time *t*. Combining Equations (3) and (4), hydrolytic damage was defined as:

(5) $$d_{h}=1-\frac{M_{nt}}{M_{n0}}=1-\frac{S_{t}}{S_{0}}=1-e^{-ut}=1-e^{kewt}$$

From Equation (5), hydrolytic damage varies from zero for the non-degraded material to one, considering that the molecular weight reaches 0. However, in the work of Vieira et al. [37], the evolution of the mechanical behavior was well predicted until 50% of hydrolytic damage. When strength decreases to half of its initial value, hydrolytic damage is equal to 50% (i.e., $d_{h}$ = 0.5). The term “half-life” applied to biodegradable structures is commonly found in literature and is an important functional design requirement. Half time is the degradation time needed to reduce the structural strength to half of its original value. Furthermore, in the current work, when the molecular weight reaches a threshold value, the element will be erased. In the current work, we will use this simple random scission model to predict the molecular weight distribution and, hence, the hydrolytic damage. Other more sophisticated models could be used to describe the polymer degradation and the heterogeneous evolution of molecular weight along the volume. Furthermore, the evolution of constitutive model parameters will be directly related to the local chemical damage, or the local molecular weight.

As mentioned previously, several factors affect the degradation rate *u*. In the case of water concentration, for large volumes, this can be computed using Fick’s Law, given by Equation (1). However, in some works, diffusion was considered instantaneous, and water concentration was considered homogeneously distributed along the volume from the beginning of the hydrolytic process. This simplifies the numerical model. On the other hand, temperature can also be considered homogeneously distributed along the volume. Since macromolecules remain macro despite their multiple random scissions [43], the concentration of ester groups, e, can also be considered uniformly distributed along the volume and constant during the initial phase of degradation.

Considering a generic geometry and boundary conditions, when loads are applied to the structure, the stress field will vary from point to point along the volume, resulting in an heterogeneous distribution of the degradation rate. The degradation rate will be a scalar field that can be related to a stress tensor invariant, such as the von Mises equivalent stress $\sigma^{\prime }$, and then, in an initial time increment, we will have $u(x,y,z,\sigma^{\prime })$. Hence, hydrolytic damage and the molecular weight in the next time increment will also vary from point to point along the volume, according to the stress field. And since the mechanical behavior, and the constitutive model parameters are directly related to the local molecular weight, an initial homogenous material will become heterogeneous on the next time increment, with some regions more degraded than others. The software must compute, in each finite element of the FEM model, at each time increment, the hydrolytic damage accumulated until then $d_{h}\left(x,y,z,u,t\right)$ based on the local degradation rate $u(x,y,z,\sigma^{\prime },w)$ that depends on the stress field, as well as local water concentration. Furthermore, the model parameters of the constitutive model, in each element, can be computed based on hydrolytic damage accumulated or the current molecular weight. On the other hand, the constitutive model parameters will affect the stress field on the next time step since the material becomes softer with a decrease in molecular weight. Hence, the degradation rate also depends on time, $u(x,y,z,t,w,\sigma^{\prime })$ and must be computed for each element and each time increment. Only if any loads are applied during degradation, or in the case of a bar having a constant cross-section with loads on the tops, will this result in a homogenous stress field that leads to homogenous degradation. If hydrolytic damage evolves homogeneously along the volume, it will only depend on time, independentease below a crit of the position.

The hydrolytic degradation regards the model developed by Wang et al. [39]. In this model, the rate of polymer chain scission is given by Equation (6)

(6) $$\frac{dR_{s}}{dt}=C_{e,0}*\left[1-\alpha \left(\frac{R_{s}}{C_{e,0}}\right)\right]*\left[k_{1}+k_{2}{\left(k_{a}\frac{C_{ol}}{m}\right)}^{0.5}\right]$$

where $R_{s}$ is the mole concentration of chain scission, $C_{e,0}$ is the initial ester bonds, $C_{ol}$ is the short chain, $K_{1}$ and $K_{2}$ are the kinetic rate constants for non-catalytic and autocatalytic hydrolysis reactions, α and β are constants indicating the nature of chain scissions, $K_{a}$ is the equilibrium constant for acid disassociation of the carboxylic and *m* is the average degree of polymerization of the short chains.

The rate of concentration of the ester bond is given by Equation (7).

(7) $$\frac{dR_{ol}}{dt}=\alpha \beta {\left(\frac{R_{s}}{C_{e,0}}\right)}^{\beta -1}\frac{dR_{s}}{dt}$$

where $R_{ol}$ is the concentration of the ester bond of all short chains.

It is known that the oligomers are capable to diffuse through the polymer, thus using Fick’s law for diffusion of the oligomer, thus for tridimensional case:

(8) $$\frac{\partial C_{ol}}{\partial t}=\frac{\partial R_{ol}}{\partial t}+\frac{\partial }{\partial x}\left(D_{K,x}\frac{\partial C_{ol}}{\partial x}\right)+\frac{\partial }{\partial y}\left(D_{K,y}\frac{\partial C_{ol}}{\partial y}\right)+\frac{\partial }{\partial z}\left(D_{K,z}\frac{\partial C_{ol}}{\partial z}\right)$$

where $D_{K,x}$, $D_{K,y}$ and $D_{K,z}$ are the diffusion coefficient in *x*, *y* and *z* directions. And, the diffusion coefficients are given by equation

(9) $$D_{K,x}=D_{K,poly}+\left(1.3\varepsilon_{x}^{2}+0.3\varepsilon_{y}^{3}+0.3\varepsilon_{z}^{3}\right)\left(D_{K,pore}+D_{K,poly}\right)$$

where $D_{K,poly}$ is the diffusion coefficients for the non-degraded polymer, $D_{K,pore}$ is the water filled pore. The eroded elements, $\varepsilon_{i}$ is given by Equation (10).

(10) $$\varepsilon_{i}=\frac{1}{2\eta_{r}}\sum_{i}S\left(i,t\right)\;;i=x,y,z$$

### 2.2. Erosion Damage

To correctly simulate the erosion of a polymer, bulk and surface erosion must be regarded, as in the work of Sevim and Pan [21]. Two rules for the erosion are regarded in this work. First, the erosion will occur when the molecular weight of an element decrease below a critical value. Second, the eroded internal element must be in contact with a previous eroded element [44]. Thus, the erosion starts from the surface in contact with water and evolves towards the interior of the part.

The initial molecular weight follows a statistical distribution. Thus, it is possible to compute the initial molecular weight, $M_{n0}$, of each element of the finite element model [21] (Equation (11)). In this work, the Weibull distribution is used once this distribution allows calibration of the distribution parameters, improving the model’s accuracy:

(11) $$M_{n0}=M_{nmean}+R\cdot M_{nsd}$$

where $M_{nmean}$ is the average molecular weight, $M_{nsd}$ is the molecular weight standard deviation, and *R* is the Weibull distribution. This distribution of molecular weight, and its standard deviation are related to the polydispersity index (PI) measured in the GPC analysis.

This erosion model results in a sudden release of oligomers when the critical molecular weight is reached. This behavior is not correct for all polymers, but it can be improved considering an incubation time. This way, oligomers will be released more progressively [21].

For some polymers, such as PLA, the erosion process starts as soon as the polymer is in contact with the surrounding environment. [21]. Thus, the decrease in volume can be described as:

(12) $$\frac{dV(t)}{dt}=-B$$

where *B* is a material constant.

Thus, when the volume decreases to zero, the respective element was deleted from the mesh.

Finally, the molecular weight is given by Equation (13)

(13) $$M_{nt}=M_{n0}\frac{1-\alpha {\left(\frac{R_{s}}{C_{e,0}}\right)}^{\beta }}{1+N_{dp,0}\left(\frac{R_{s}}{C_{e,0}}-\frac{\alpha }{m}{\left(\frac{R_{s}}{C_{e,0}}\right)}^{\beta }\right)}$$

where $N_{dp,0}$ is the degree of polymerisation.

Knowing the polymer density and the element volume, the total mass loss can be obtained regarding the contributions of short-chain diffusion, erosion, and hydrolytic damage.

(14) $$M_{loss_{t}otal}=M_{n0}*M_{d}*M_{hd}+M_{e}$$

where $M_{loss_{t}otal}$ is the total mass loss, $M_{d}$ is the mass loss due to diffusion (Equation (15)), $M_{e}$ is the mass loss due to erosion, and $M_{hd}$ is the mass loss due to hydrolytic damage that depends on the stress field (Equation (16)).

(15) $$M_{d}=\frac{1-\alpha {\left(\frac{R_{s}}{C_{e,0}}\right)}^{\beta }}{1+N_{dp,0}\left(\frac{R_{s}}{C_{e,0}}-\frac{\alpha }{m}{\left(\frac{R_{s}}{C_{e,0}}\right)}^{\beta }\right)}$$

(16) $$M_{hd}=e^{-ut}$$

The proposed model regards the contribution of the hydrolytic damage and erosion model proposed by [21] with the effect of the stress field on the degradation proposed by [36].

### 2.3. Constitutive Model

In this work, we will consider a large-strain monotonic loading case. Hence, we will use two time-independent constitutive models. Namelly, the well-known $J_{2}$ plasticity model, with isotropic hardening and the Neo Hooke hyperelastic model were applied, and results from both models were compared. Thus, it is possible to observe the difference between both material models when used to simulate hydrolytic damage, erosion due to diffusion and the mechanical behavior of a biodegradable polymeric structure after a given degradation time. The use of a time-dependent constitutive model leads to three different time histories associated with the coupling between mechanical loading, diffusion, and hydrolytic degradation, which are then integrated with a finite element (FE) formulation in order to solve the time-dependent boundary value problem. The internal variable to describe the hydrolytic damage, $d_{h}\left(x,y,z,u,t\right)$, was introduced as a scalar field to degrade the material’s elastic properties, which depends on another scalar field, the degradation rate $u(x,y,z,\sigma^{\prime },w)$, that in turn depends on the stress field and water concentration field.

#### 2.3.1. Hyperelastic Model

Many recent works have aimed to describe the mechanical behavior of biodegradable polymers during degradation based on the neo-Hookean hyperelastic models, as the works of Soares et al. [35]. As with most hyperelastic models, the neo-Hookean one can be applied to describe the relaxed configuration presented by polymers after loading, especially in the case of polymers with a more ductile and nonlinear mechanical behavior, such as elastomers. Thus, neo-Hookean models can represent with good precision the hardening and softening behaviors that give the S-shaped stress-strain curves of some polymers.

Usually, the hyperelastic properties of polymers are represented in terms of the strain energy density (*W*) and can be expressed by the Kirchhoff stress tensor ($\tau_{ij}$) shown in Equation (17):

(17) $$\tau_{ij}=F_{ik}\frac{\partial W}{\partial F_{kj}}$$

where F is the deformation gradient given by $ABAQUS^{TM}$. Furthermore, spatial forms of constitutive relations are often written in terms of the invariants of the left Cauchy–Green deformation tensor. Equation (18) shows the left Cauchy–Green deformation tensor B:

(18) $$B_{ij}=F_{ik}F_{jk}$$

However, many polymeric materials are nearly incompressible. Thus, the distortions in configuration are better described by the deviatoric left Cauchy-Green tensor ($\bar{B}$) exhibited in Equation (19):

(19) $${\bar{B}}_{ij}=B_{ij}-\frac{1}{3}B_{kk}\delta_{ij}$$

where $\delta_{ij}$ is the Kronecker delta. Thus, the invariants can be written in terms of $\bar{B}$. However, for this hyperelastic implementation, only the first invariant ($I_{B}$) will be necessary. It is described by Equation (20), and its deviatoric form by Equation (21):

(20) $$I_{B}=\mathrm{tr}\left(B_{ij}\right)$$

(21) $$\bar{I_{B}}=J^{-2/3}I_{B}$$

where *J* is the Jacobian of the deformation defined by $J=\det F_{ij}$ which represents the volume change (J = 1 for incompressible material). Based on the first invariant, a common representation for the strain energy density of the neo-Hookean model is:

(22) $$W\left(\bar{I_{B}},J\right)=C_{10}\left(\bar{I_{B}}-3\right)+\frac{1}{d}{\left(J-1\right)}^{2}$$

where $C_{10}$ is the material parameter associated to distortion in configuration meaning it is linked to the shear modulus (μ), and *d* is the material parameter related to volume change (d=0 for incompressible material) meaning it is related to the the bulk modulus (*k*).

(23) $$C_{10}=\frac{E}{4(1+\nu )}$$

(24) $$d=\frac{6(1-2\nu )}{E}$$

Thus, by applying Equation (22) in Equation (17) and calculating the derivative, the Kirchhoff stress can be written in terms of the left Cauchy–Green tensor as shown in Equation (25):

(25) $$\tau_{\mathrm{ij}}=\frac{2C_{10}}{J^{2/3}}\left(B_{ij}-\frac{1}{3}B_{kk}\delta_{ij}\right)+2d\left(J-1\right)\delta_{ij}$$

As this work aims to investigate the hydrolytic damaged caused in the polymer, the $C_{10}$ parameter will be function of the hydrolytic damaged ($d_{h}$) as proposed by Taguti et al. [36]. Finally, as $ABAQUS^{TM}$ operates with the Cauchy stress tensor ($T_{ij}$), it is mandatory to convert the Kirchhoff stress to the Cauchy one by dividing the Kirchhoff tensor by the Jacobian ($T_{ij}=\tau_{ij}/J$).

#### 2.3.2. Elastoplastic Model

The stress-strain behavior of the elastoplastic model was built in two parts. First of all, the material goes through an elastic stage governed by Hooke’s law, then a plasticity stage following a $J_{2}$ Mises plasticity with piecewise-linear isotropic hardening. Thus, the elastic part is represented by the Cauchy stress tensor shown in Equation (26):

(26) $$T_{ij}=2\mu \varepsilon_{ij}+\lambda \varepsilon_{kk}\delta_{ij}$$

where λ is the Lame parameter defined by λ=2νμ/[1−(2ν)]. By adopting the Von Mises equivalent stress ($\bar{\sigma }$) and applying it to the yield function (*f*), the yield test fails if f>0:

(27) $$f=\bar{\sigma }-Y_{old}$$

where ($Y_{old}$) is the yield strength from the last step (it is constant while in elasticity). Furthermore, if the material has yielded, the calculation of the equivalent plastic strain begins following Equation (28):

(28) $$d\bar{\varepsilon_{p}}=\frac{\bar{\sigma }-Y_{old}}{3\mu +H}$$

where $d{\bar{\varepsilon }}_{p}$ is the increment for the equivalent plastic strain and *H* is the tangent modulus of the material’s stress-strain curve. The yield strength shall be updated in order to satisfy Equation (27).

(29) $$Y_{new}=Y_{old}+Hd\bar{\varepsilon_{p}}$$

The deviatoric (${\sigma^{\prime }}_{ij}$) and hydrostatic (${\sigma^{h}}_{ij}$) stress components were calculated in order to update the $T_{ij}$. Furthermore, a multiplier factor (Λ) was applied to update the deviatoric stress as shown in Equation (31).

(30) $$\Lambda =\frac{Y_{new}}{Y_{new}+3\mu d\bar{\varepsilon_{p}}}$$

(31) $$T_{ij}=\Lambda {\sigma^{\prime }}_{ij}+{\sigma^{h}}_{ij}$$

Finally, in order to apply the hydrolytic damage, a $d_{h}$ factor was applied to the μ of the material, which represents what would happen to the material under submersible applications.

### 2.4. Finite Element Implementation

This work will investigate the effect of erosion and hydrolytic damage on biodegradable polymers using a generic constitutive model. We also want to understand how the choice of the constitutive model affects the finite element simulation results. Thus, a User Material for Explicit simulations (VUMAT) linked to the finite element software $ABAQUS^{TM}$, was implemented to model the hydrolytic damage proposed by Taguti et al. [36] and the erosion model proposed by Thombre and Himmelstein [45].

The geometric model to simulate polymer degradation was a parallelepiped having the following dimensions 20 mm × 20 mm × 70 mm. This model is relatively thick to allow the analysis of the interaction between erosion and hydrolytic damage. This finite element model uses 3500 eight-node reduced integration brick elements (C3D8R in $ABAQUS^{TM}$ terminology). Also, an open hole model with same basic dimensions and a hole with a 10 mm radius was also used. This last model also used the same element type as the previous model, but this model has 3250 elements.

**Figure 1:**
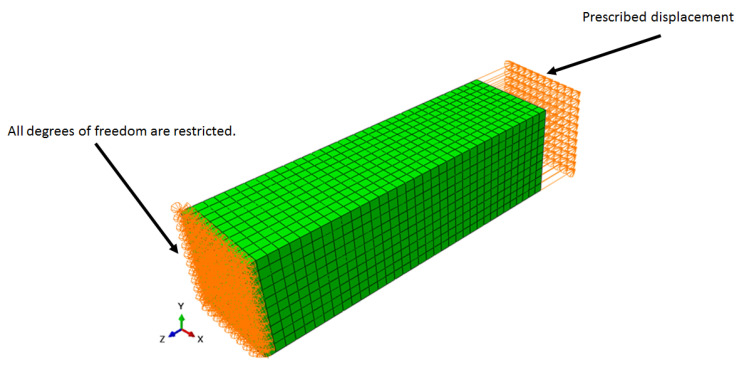
Model 1 boundary conditions.

**Figure 2:**
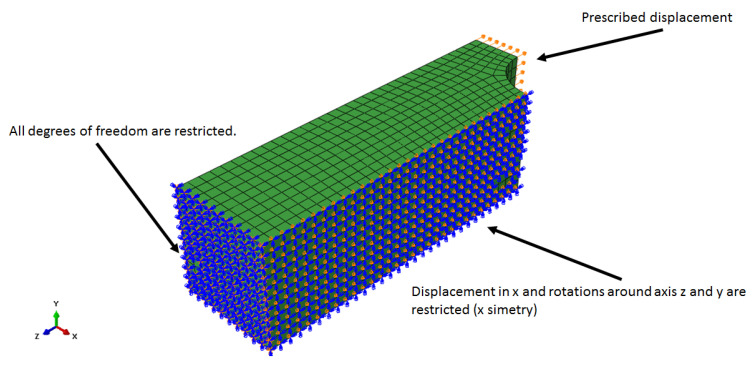
Model 2 boundary conditions.

The boundary conditions are presented in Figure 1 and Figure 2, where on the left side, all the degrees of freedom are restricted, and on the other side, a prescribed displacement of 0.1 mm was applied in *z* direction for the first configuration, and, for the second configuration, the symmetry condition along the *x*-axis is applied (*x* displacement and rotations around *y* and *z* axis are restricted).

The different models lead to different stress states, allowing to assess the influence of the stress level on the polymer degradation.

**Figure 3:**
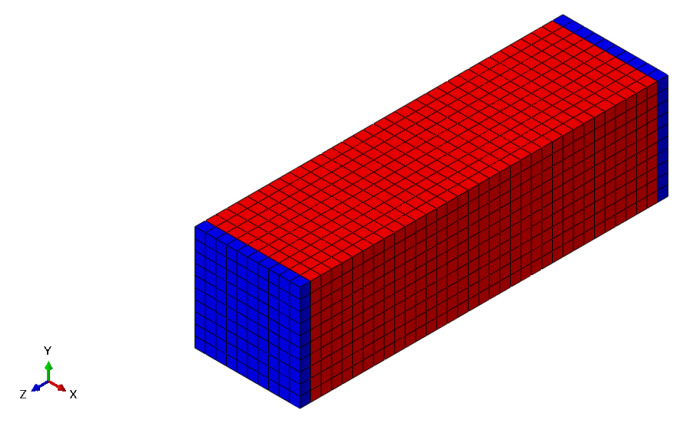
Model surface elements.

**Figure 4:**
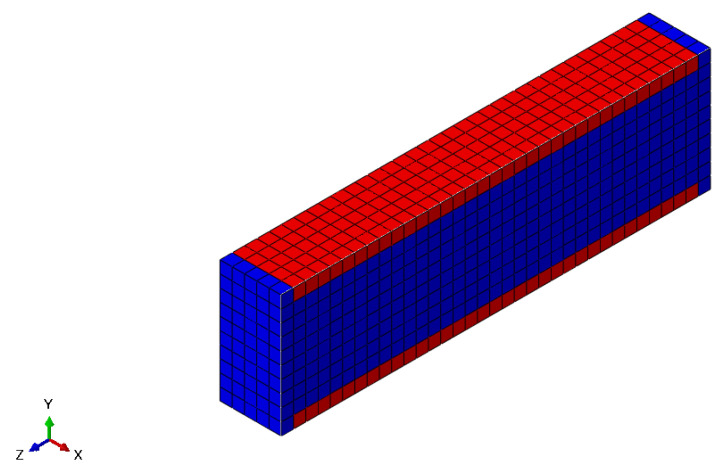
Half cut view of the model surface elements.

As the damage process starts at the surface, it is important to select only the surface elements of the finite element model, as presented in Figure 3 and the half cut view in Figure 4.

To avoid element deletion in the region where the boundary conditions are applied, the left and right sides of the model are not allowed to degrade. This strategy ensures that the restrictions remain the same during simulation time and helps avoid problems that can occur in explicit simulations.

**Figure 5:**
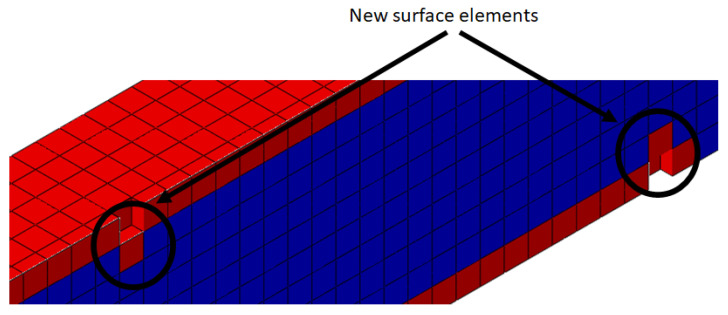
New surface elements.

As mentioned before, when the molecular weight decreases below a critical value, the element was deleted, thus an internal element turns into a surface element, as is presented in Figure 5.

A non-local methodology was used to alleviate the effect of the model element size to avoid localization issues, thus, the hydrolytic damage (Equation (5)) was averaged regarding the values of the nearby elements (Equation (32)).

(32) $$dh\left(x\right)=\int_{V}\alpha \left(x,y\right)dh\left(y\right)dV$$

where dh(x) is the non-local hydrolytic damage, α(x,y) is the non-local average operator ([46]) and dh(y) is the local hydrolytic damage. Equations (33) and (34) are used to obtain the non-local operator.

(33) $$\alpha \left(x,y\right)=\left(\frac{\alpha_{0}\left(x,y\right)}{\int_{V}\alpha_{0}\left(x,y\right)dV}\right)$$

(34) $$\alpha_{0}\left(x,y\right)={\left(1-\frac{\left(x-y)(x-y\right)}{L_{r}}\right)}^{2}$$

where $L_{r}$ is a scalar related to the intrinsic length, which is defined as the greatest possible distance from a material point influencing the hydrolytic damage to a material point x.

The Weibull distribution probability density function (PDF) was used for the initial molecular weight distributions over the elements of the model. The probability of the value of parameter R, which multiplies the $M_{nsd}$ varying from “a” to “b” is given by Equation (35).

(35) $$Pr\left[a\leq R\leq b\right]=\int_{a}^{b}f\left(x\right)dx$$

**Figure 6:**
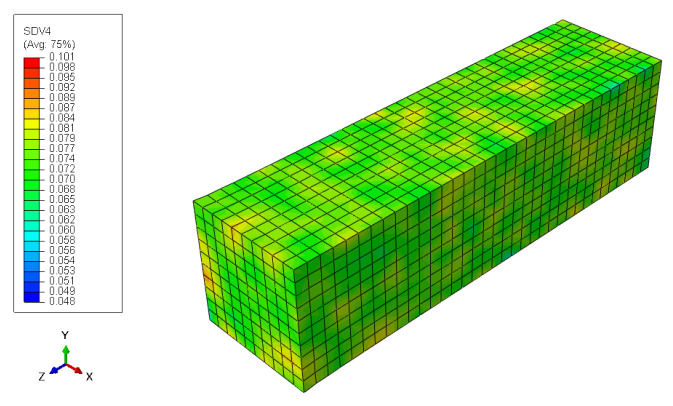
Finite element model molecular weight distribution.

In this way, the value of R is computed according to a Weibull distribution, given by Equation (36).

(36) $$\left(x:\psi ,\gamma \right)=\left\{\begin{array}{cc} \frac{\gamma }{\psi }{\left(\frac{x}{\psi }\right)}^{\gamma -1}e^{{\left(-x/\psi \right)}^{k}} & x\geq 0\\ 0 & x<0 \end{array}\right.$$

where γ and ψ are dimensionless parameters of the Weibull distribution that determine the heterogeneity of the initial molecular weight (Equation (11)) and can be determined experimentally by GPC, where the polydispersity index (PI) is a measure of the heterogeneity of the molecular weight distribution. For instance, the non-homogeneous distribution of molecular weight in the finite element model is shown in Figure 6.

As mentioned before, the Weibull distribution could assume several probability density distribution shapes, just changing its parameters γ and ψ. On the other hand, these parameters must be adjusted to correctly fit the experimental data. This approach was used by Saconi et al. [47] to model the magnesium alloy pitting corrosion. For this work, we assume that the Weibull parameters are ψ=0.5 and γ=2.0.

## 3. Results

This section presents practical examples to simulate polymer degradation, accounting for the effects of the erosion model from Sevim and Pan [21] and the hydrolytic damage model from Taguti et al. [36]. These approaches were implemented together as an $Abaqus^{TM}$ user material subroutine for explicit simulations.

**Figure 7:**
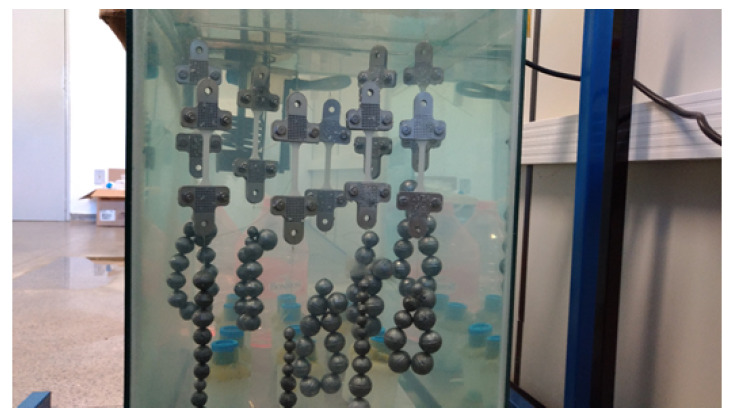
Degradation experiment of biogradable polymers regarding different stress fields [36].

The simulations require some material properties, such as Young modulus, Poison coefficient, erosion rate, degradation rate, and hydrolytic damage evolution law. Vieira et al. [37] performed several experiments to characterize the mechanical properties of a PLA-PCL blend. To measure the hydrolytic damage under the influence of the stress field, an experimental setup such as the one shown in Figure 7 can be used.

**Figure 8:**
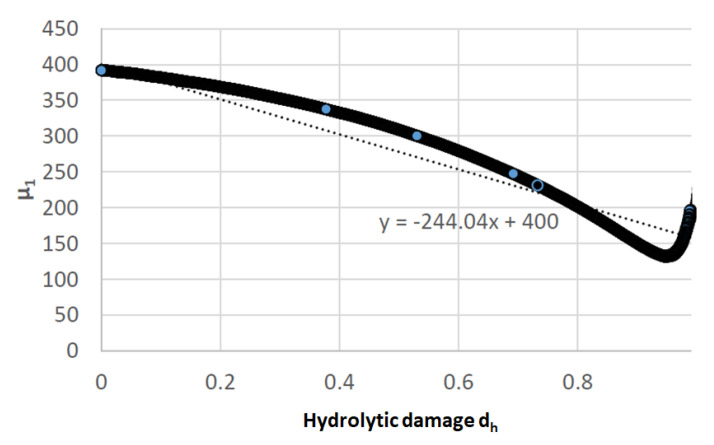
Experimental hydrolytic damage $d_{h}$ [48].

The material elastic properties were E=824 MPa for Young modulus, and ν=0.33 for the Poison coefficient [48]. Also, from the same reference, the degradation test results presented the relation between the hydrolytic damage ($d_{h}$), and the shear modulus ($\mu_{1}$) as seen in Figure 8.

**Figure 9:**
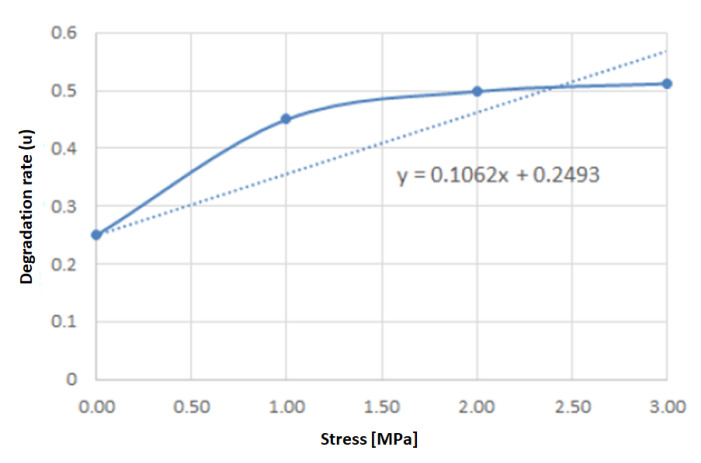
Degradation rate *u* [48].

The degradation rate (*u*) used in this work was obtained from the experimental data from Vieira et al. [37], which the authors Taguti et al. [36] assumed a linear relationship with the stress (Figure 9). Also, for the hydrolytic degradation rate, the following relation was adopted: $u=0.1062\sigma_{vM}+0.2493$ [36], where $\sigma_{vM}$ is the von Mises stress.

In addition, the volumetric erosion rate (*B*) for the erosion model (see Equation (12)) adopted was 0.65 m${}^{2}$/week [21]. Moreover, the critical molecular weight adopted was $M_{n,critical}=2.7\times$ 10${}^{4}$ g/mol. The other parameters used in this work are: $D_{K,poly}=5.0\times 10^{-15}$ m${}^{2}$/week, $D_{K,pore}=1.0\times 10^{-5}$ m${}^{2}/$week, $M_{nmean}=7.5\times 10^{4}$ g/mol, $M_{nsd}=2.0\times 10^{4}$ g/mol, $k_{1}=5.0^{-6}$ 1/week, $k_{2}=2.0^{-2}$ m${}^{3}$/(mol × week), m=4, α=0.4, β=1, $k_{a}=1.35\times 10^{-4}$.

The first case is the stress-free simulation. It is important to mention that, as this is a model regarding different degradation mechanisms, implemented in a 3D finite element model of a generic geometry, the lack of experimental results for this case made it difficult to perform a direct comparison between simulations and available experimental results. On the other hand, the degradation time for a stress-free case is proportional to the results presented by Sevim and Pan [21], where, for a specimen with dimensions of 20 × 10 × 2 mm, it took almost 30 weeks for a 100% of mass loss. For this work, a 20 × 20 × 70 mm finite element model took 90 weeks to reach 100% of mass loss (Figure 11).

**Figure 10:**
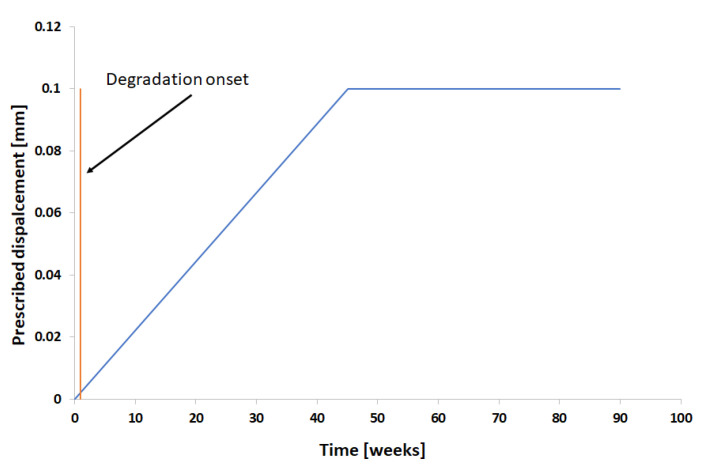
Prescribed displacement schema for increasing load case.

Furthermore, the second case involved the verification of the effect of a stress field in the degradation process by applying the hyperelastic material model allied with the proposed degradation model. As mentioned before, a prescribed displacement was imposed in one of the extremities, and the displacements were restricted in the other extremity, as shown in Figure 1. The prescribed displacement follows the profile shown in Figure 10, where the prescribed displacement increases until reaching 0.1 mm at the middle of the simulation step, then the displacement is kept constant until the end of the analysis.

**Figure 11:**
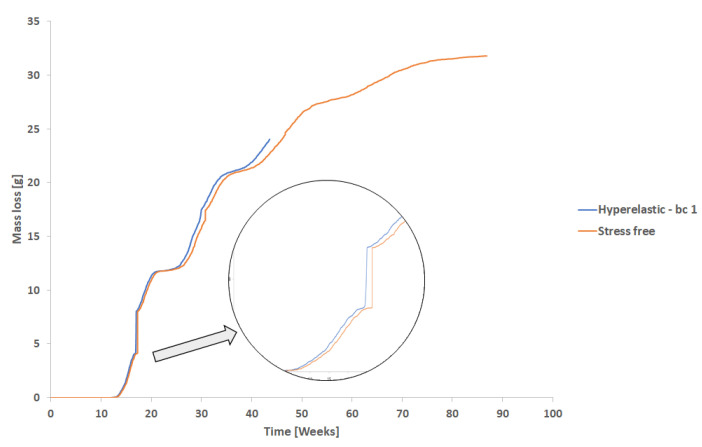
Hyperelastic model with low stress versus stress-free.

In this case, degradation starts at 0.9 weeks with a very low load. In this part, the stress field accelerates the degradation but the difference for the stress-free condition is not very significant, as is shown in the detail in Figure 11. It is important to mention that the proposed model affects not only the model geometry, due to element deletion, but also the material’s elastic properties.

A third case was also studied to assess the effect of the material model adopted, the same simulations in the second case were performed regarding the elastoplastic behavior. Within the low-strain domain, the response of elastoplastic and hyperelastic materials are similar. On the other hand, these modeling strategies differ significantly for large strains, thus it is worth investigating this effect on the degradation model.

For both the second and third cases, as the displacement increases, as well as degradation, the mass loss reduces the cross-section of the model increasing the stress and the degradation rate. After 90 weeks, the high stress due to the reduction of the cross-section leads to the rupture of the model (end of the curve for the hyperelastic model in the Figure 11).

**Figure 12:**
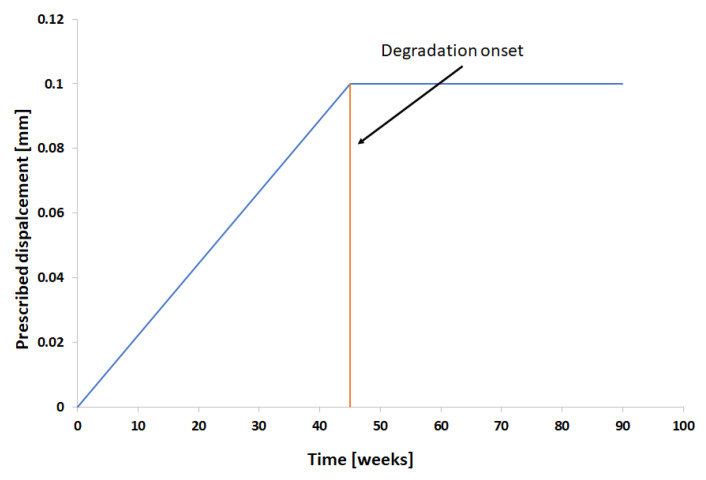
Prescribed displacement schema for constant prescribed displacement.

**Figure 13:**
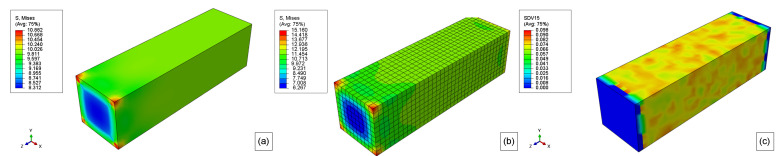
(**a**) Initial hyperelastic von Mises stress field, (**b**) initial elastoplastic model Stress field, and (**c**) initial (0 weeks) molecular weight field for stress-free.

For the rest of the simulations, the degradation only starts after the prescribed displacement reaches 0.1 mm, and then it is kept constant (Figure 12). This displacement results in a stress field as show in Figure 13a,b. Thus, the start of the degradation was also the start of time counting (0 weeks).

The von Mises stress field for boundary conditions 1 is shown in Figure 13b. For this condition, the elastoplastic model stress reaches a maximum of 14.119 MPa, which is within the elastic limit for this material. Also, the stress for the majority of this model is around 12 MPa.

The initial molecular weight distribution is shown in Figure 13c. This distribution follows Equation (11). Once again, it is important to mention that the model extremities will not be degraded to avoid noise in the response of the explicit simulations as the boundary conditions are applied in these locations.

As the stress field affects the hydrolytic damage, the same generic model is simulated regarding a different boundary condition, as shown in Figure 2. The prescribed displacement also follows the pattern show in Figure 12. Thus, Figure 13b shows the von Mises stress field for this case. In this case, the maximum von Mises stress is 14.272 MPa on the corners of the model, but for most of the model, the stress is around 12 MPa as for boundary condition 1.

**Figure 14:**
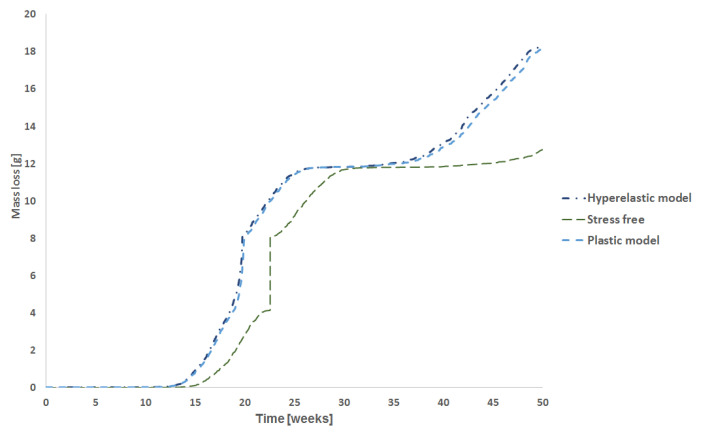
Mass loss vs time for the stress-free condition, the elastoplastic model and the hyperelastic models.

Figure 14 shows the mass loss over time for the stress-free condition and for hyperelastic and elastoplastic models. For this case, after 50 weeks of degradation time, the stress-free model had lost approximately 13 g (around 37%) of mass, which is coherent for this degradation time regarding the experiments [21].

The molecular weight in Figure 13c is the same for all cases (elastoplastic and hyperelastic cases), once the Weibull distribution parameters did not change. As mentioned before, the model extremities were not allowed to degrade, thus the elements that belong to those regions did not go into the degradation loop inside the material model. As a result of this procedure, those regions appear as if they not have a molecular weight (blue regions in the extremities of the figures).

**Figure 15:**
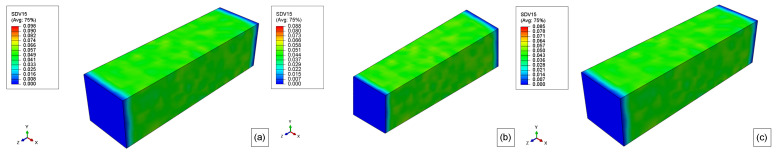
10 weeks molecular weight field for (**a**) stress-free, (**b**) elastoplastic, and (**c**) hyperelastic model.

**Figure 16:**
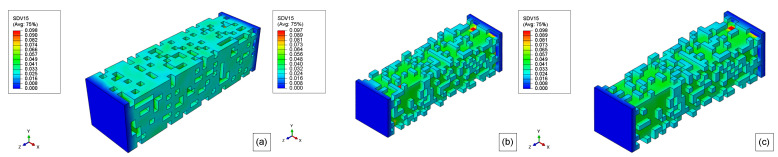
20 weeks molecular weight field for (**a**) stress-free, (**b**) elastoplastic, and (**c**) hyperelastic model.

**Figure 17:**
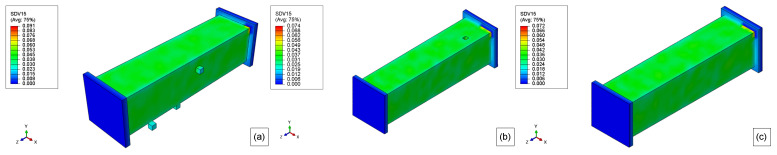
30 weeks molecular weight field for (**a**) stress-free, (**b**) elastoplastic, and (**c**) hyperelastic model.

**Figure 18:**
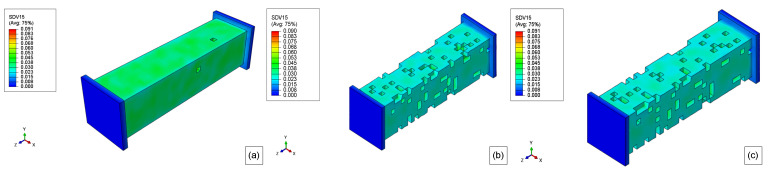
40 weeks molecular weight field for (**a**) stress-free, (**b**) elastoplastic, and (**c**) hyperelastic model.

**Figure 19:**
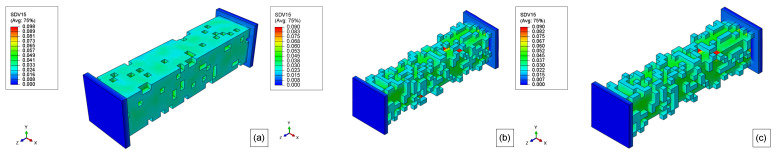
50 weeks molecular weight field for (**a**) stress-free, (**b**) elastoplastic, and (**c**) hyperelastic model.

The results of the molecular weight of PLA-PCL degradation for 10 weeks, 20 weeks, 30 weeks, 40 weeks, and 50 weeks are presented in. Figure 15, Figure 16, Figure 17, Figure 18, and Figure 19, respectively. Where the labels a, b, and c of these figures represent the stress-free, elastoplastic, and hyperelastic conditions, respectively.

As expected, the presence of load increases the model mass loss, and the higher the load, the higher the differences compared to the stress-free condition (Figure 11 and Figure 14). Additionally, the material behavior adopted can affect the amount of mass loss, mostly for higher stresses. Exceptionally, for this case, the difference in boundary conditions did not affect the degradation behavior once those differences were not significant in terms of stress intensity.

After 10 weeks, the reduction of the molecular weight is very clear compared with the initial condition in Figure 13c, but none of the model elements had reached the critical value for both boundary conditions.

10 weeks later (20 weeks), several surface elements had reached their critical values, $M_{n,critical}$, and they are deleted from the model (Figure 16). Additionally, the molecular weight, $M_{n}$, of almost all elements is getting close to the critical value in Figure 16a. For the loaded models, almost all the surface element was eroded as seen in Figure 16b,c. Thus the second layer of elements, which was not in contact with the exterior environment, forms a new surface and then starts the degradation of these elements. Therefore, the influence of the load in the mass loss of the models is explicit when comparing the unloaded case to the loaded ones. Additionally, the original surface has more elements than the internal newer surfaces. Also, the elastoplastic and hyperelastic models show quite similar results, but not exactly the same.

For the non-stress condition, the 30th week shown in Figure 17a, almost all the surface elements were completely eroded, and the mass loss was very severe for this model geometry and degradation time (see Figure 11). When the model reaches its 30th week, all the first layers of elements were deleted, and as the degradation started to run for this layer, the model almost had not lost mass during the 27th week till 34th week (almost flat plateau in Figure 14).

In Figure 18a, there are no more elements from the original surface, and as the degradation time, for a particular element, starts to run only when this element becomes a surface element, most of these second layer elements have been degraded for 10 weeks (some elements have been degraded for more time), thus the mass loss rate during this week is very low. This slower erosion was seen for the loaded cases since their outer layer was eroded first (see Figure 11). Thus, for the loaded cases in the 40th week of degradation, as the molecular weight of the surface elements reaches its critical value, the mass loss accelerates again but at a lower rate than before since there are fewer elements in the second layer compared to those in the first layer, considering that the dimension of the model are decreasing as shown in Figure 18b,c. The differences between the elastoplastic and hyperelastic models are almost indistinguishable.

Finally, after 50 weeks (Figure 19), the erosion rate for the unloaded case starts to accelerate again as the surface elements are reaching their critical value, as it can be related to Figure 11. Regarding the loaded cases, when the model reaches the 50th week several elements have been deleted from the model after reaching its critical value. This reduction of the cross-section increases the stress and, as consequence, the degradation rate, thus the mass loss rate increases by around 48th week, on the other hand, as this surface has fewer elements than the first layer, the increase in the mass loss is not so substantial than for the 19th week, additionally, the elements of the second-layer did not start to degrade at the same time as for the first layer. The hyperelastic stress field from boundary condition 2 is different from boundary condition 1 as seen in Figure 19c.

**Figure 20:**
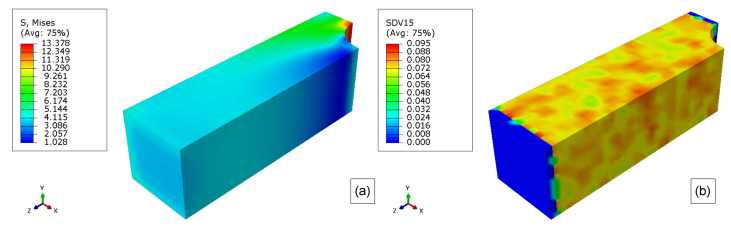
(**a**) Open hole von Mises stress field and (**b**) initial molecular weight distribution.

As mentioned before, the stress field accelerates the polymer degradation. Thus, for the open hole model, the stress gradient is significant due to the stress concentration near the hole as seen in Figure 20a, and the mass loss is therefore faster for this model. It is important to mention, that the hyperelastic model was applied for these simulations. Figure 20b shows the initial molecular weight distribution for the open hole model. Due to the new mesh for this model, the molecular weight distribution is different from the previous model once the Weibull distribution generates different values for the mesh elements.

**Figure 21:**
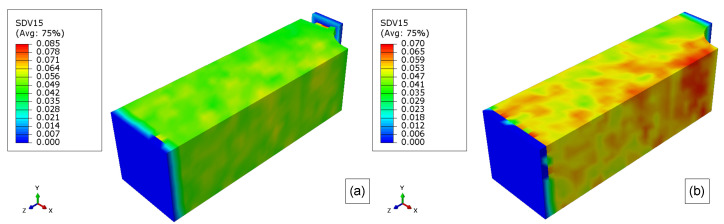
(**a**) Rupture of the model and (**b**) molecular weight distribution after 10 weeks.

After 10 weeks, the rupture occurs in the high stress region as shown in Figure 21a as the reduction of molecular weight in the region of the hole is significant as exhibited by Figure 21b.

## 4. Discussion

There are only few numerical approaches to simulate the mechanical behavior that consider the effect of volume decrease due to erosion and material softening due hydrolytic damage. This work is original in combining these two types of approaches. On one hand there is a decrease of strength and an evolution of the stress field due to volume decrease resulting from erosion. Such as in the work of Sevim and Pan [21], elements are deleted from FEM model when molecular weight reaches a threshold value. However, in the work of Sevim and Pan [21] the elastic properties of the remaining volume to not vary. On the current approach, on other hand, the remaining volume also changes its elastic properties, becoming softer as hydrolysis progresses, and the material parameters of the constitutive model evolve as function of the molecular weight evolution. In the work of Taguti et al. [36], the volume remains the same and only the material parameters evolve along the volume. In the current work, as in the work of Taguti et al. [36], the rate of molecular weight evolution depends on the effect of stress field. Therefore it also affects erosion, since in regions of high stress concentration the molecular weight threshold value is reached faster. Since, in most applications of biodegradable polymers, the parts are not in a stress-free condition, this model allows to predict the behavior of biodegradable polymer for real applications. Considering that time independent constitutive models were used in the present work, it enable to simulate quasi-static loading case scenarios.

For those applications, the life time due hydrolysis is an important functional requirement that must be adressed during the design phase, and the use of finite element method is a very powerful tool to accomplish the part design. This numerical approach contributes to the modeling of biodegradable polymeric parts.

The simulation results of this numerical approaches, for the stress-free condition, are in accordance with the experimental data found in literature for PLA, despite the differences in geometry and boundary conditions. For example, the model presented in this work, took almost 90 weeks to complete erosion, and 30 weeks for the work presented by Grizzi et al. [16]. These differences are due to model geometry (smaller for Grizzi et al. [16]) and the Weibull parameters used for the initial molecular weight distribution must be experimentally calibrate.

Another remarkable point from our results, is that there are some very high erosion rates around 20 weeks, when several elements of the first layer were eliminated almost together. This high erosion rate was also presented in some experiments as performed by Grizzi et al. [16], where this high erosion rate occur around 12 weeks, considering that the test specimen was 3 mm thick.

When the model was loaded, degradation and erosion accelerated for the case with low stress (Figure 11). It is important to mention, that erosion affects the part load bearing capacity due to mass loss (reduction of cross sectional area) but also degradation affects material strength and the elastic properties of the material. Thus, as the stress level is low, degradation and erosion rates are very similar to that found for the stress-free condition. As the load increases, as well as the time, the mass loss also increases, resulting in a reduction of the cross-section area. This reduction, increases stress, accelerating the mass loss process and after 40 weeks, the model fails.

In a real situation load is applied from the beginning, thus it is more realistic to consider this situation. For this case, the mass loss starts 3 weeks earlier, and the rate of mass loss is greater than the case without load. Again, after 5 weeks, almost all elements of the first layer were eroded and the rate of mass loss increases.

When an elements at the surface are deleted from the mesh, internal elements become surface elements (Figure 5), and the degradation starts for this specific elements. When this happens for several elements, the mass loss slows until the deletion criteria is reached again. The plateau in the Figure 11 and Figure 14 shows this effect.

Finally, the constitutive law to model the material behavior, also affect the simulation of mass loss. For low stress regions, the hyperelastic model and the elastoplastic model almost have the same results. On the other hand, as stress increases, the effect of plasticity decreases the rate of mass loss.

## 5. Conclusions

Among the most important mechanisms of polymer degradation, hydrolytic damage and erosion have an important role in the understanding of the mechanical behavior of the biodegradable polymers over time.

Also, the effect of the stress field on the degradation of biodegradable polymers, must be accounted to improve the predictions for the parts that are used to bear loads. Thus, the proposed model, unlike other approaches, regards those important mechanism to simulate the degradation of biodegradable polymers parts that are used to bear loads.

The results shown that the effect of load on degradation rate is significant, speeding up the mass loss and leading to premature failure, as it affects not only the model geometry by element deletion, but also the elastic properties of the remaining polymer. Additionally, the mechanical behavior of the material model could affect the mass loss for high stress.

Finally, the proposed model is capable to simulate biodegradable polymers parts regarding some relevant degradation mechanisms, improving the simulation to predict the life span of parts made of biodegradable polymers.

## Data Availability

Not applicable.
